# Perceived Parental Monitoring and Health Risk Behavior among Public Secondary School Students in El Salvador

**DOI:** 10.1100/tsw.2006.284

**Published:** 2006-12-28

**Authors:** Andrew E. Springer, Shreela Sharma, Alba Margarita de Guardado, Francisco Vázquez Nava, Steven H. Kelder

**Affiliations:** ^1^ Michael and Susan Dell Center for Advancement of Healthy Living, University of Texas Health Science Center at Houston School of Public Health, Houston, TX, USA; ^2^ Department of Health and Human Performance University of Houston, Houston, TX, United States; ^3^ Save the Children, San Salvador, El Salvador; ^4^ Facultad de Medicina, Universidad Autónoma de Tamaulipas, México and Unidad de Investigación en Epidemiología Clínica, Hospital General No. 6 Instituto Mexicano del Seguro Social. Ciudad Madero, Tamaulipas, México

**Keywords:** adolescence, risk behavior, parenting, El Salvador, drug use, sexual bahavior

## Abstract

Although parental monitoring has received considerable attention in studies of U.S. adolescents, few published studies have examined how parents' knowledge of their children's whereabouts may influence health risk behaviors in adolescents living in Latin America. We investigated the association between perceived parental monitoring and substance use, fighting, and sexual behaviors in rural and urban Salvadoran adolescents (n = 982). After adjusting for several sociodemographic covariates, multilevel regression analyses indicated that students reporting low parental monitoring were between 2 to 3.5 times more likely to report risk behaviors examined. The promotion of specific parenting practices such as parental monitoring may hold promise for reducing adolescent risk behaviors in El Salvador.

### INTRODUCTION

Families living in El Salvador face several threats to the health and well-being of their children, including a poverty rate that affects 34.6% of the population[1] and among the highest homicide rates for young people in the Americas[2]. While many health threats are rooted in structural conditions that go beyond the control of families, parents may be able to reduce some health and social threats through the types of parenting practices in which they engage with their children. Parental monitoring — parents' knowledge of their children's whereabouts and activities with companions — has been found to be inversely associated with substance use[3], sexual risk behavior[4], and violence and other risk behaviors[4] in studies conducted in the U.S. Although evidence exists for an association between dimensions of parental monitoring and tobacco use in youth from Central America[5], few studies have explored the association of parental monitoring with other health risk behaviors in Central American youth. This study investigated the association of perceived parental monitoring and substance use, aggressive behavior, and sexual behaviors in a sample of rural and urban public secondary school students from the central region of El Salvador.

## METHODS

### Sample and Procedures

A secondary analysis was conducted of cross-sectional data from 982 public secondary school students attending 16 public schools located in a central department (state) of El Salvador. Data were collected in 1999 using a self-administered questionnaire and were based on a multistage, probabilistic sampling design stratified by urban and rural geographic classification in which school districts, schools, and classrooms were randomly selected. The study methods are described in greater detail elsewhere[6].

### Measures

Youth risk behavior measures were based on the U.S. Youth Risk Behavior Survey from the Centers for Disease Control and Prevention. We examined risk behaviors related to substance use, aggression, and sexual health. Substance use was assessed by three variables: episodic heavy alcohol "binge" drinking (having drunk five or more alcoholic drinks in a row on one or more days in past 30 days), lifetime cigarette use (having ever smoked a cigarette), and lifetime illicit drug use (having ever used marijuana, cocaine, or inhalants). Aggressive behavior was assessed as having engaged in a physical fight on one or more occasion in the past 12 months. Sexual/reproductive behavior was assessed by two items: ever having had sexual intercourse and having used condoms at last sexual intercourse. All risk behavior variables were dichotomized (yes/no) for the purpose of this study.

Perceived parental monitoring was assessed by two items that asked students to rate, on a five-point Likert-type scale, how often their parents know where they are during the week when not in school and during the weekend. Response choices ranged from "never" to "always". For the purpose of this study, we combined the two items to create one overall measure of parental monitoring. The composite variable was recoded to create a dichotomous measure of parental monitoring, with categories of "high" ("always" and "most the time") and "low" ("sometimes", "seldom", and "never").

### Data Analysis

Data were analyzed using Stata 9.0 (College Station, TX). Descriptive statistics and chi-square tests were conducted to describe the prevalence of parental monitoring and assess potential differences by sample characteristics. Phi coefficients were computed to evaluate the level of association between risk behavior variables. Multilevel logistic regression modeling using the "gllamm" package[7] in Stata was used to examine the association between parental monitoring and the health risk behaviors, taking into account the nesting of students within schools. The regression analyses adjusted for the following covariates: gender, age, subjective socioeconomic status, academic performance, and rural/urban geographic school location (see Table 1 for description). Significance level was set at .05.

## RESULTS

Students had a mean age (SD) of 15.25 (1.42) years. Approximately half of the students were female and half were from rural areas (see Table 1). Males, older students, and below average academically performing

**Table 1.** Prevalence (%) of high parental monitoring by descriptive characteristics, public secondary school students from central El Salvador, 1999. (n = 982)

| | Sample composition % | High parental monitoring[c] % | $\chi^2$ |
|---|---|---|---|
| *Gender* | | | |
| Female | 47.4 | 92.4 | 7.11** |
| Male | 52.6 | 86.8 | |
| *Age (in years)* | | | |
| 12 to 14 | 29.9 | 92.1 | 8.21* |
| 15 to 16 | 47.6 | 90.3 | |
| 17 to 20 | 17.7 | 83.2 | |
| *Subjective economic status* [a] | | | |
| High | 59.1 | 89.3 | 0.003 |
| Low | 40.9 | 89.2 | |
| *Geographic school location* | | | |
| Rural | 50.1 | 91.5 | 3.48 |
| Urban | 49.9 | 87.6 | |
| *Academic performance* [b] | | | |
| Better than average | 35.0 | 92.9 | 19.02*** |
| Average | 55.8 | 90.2 | |
| Below average | 9.2 | 75.4 | |

*p<.05, **p<.01, ***p<.001

[a]Subjective economic status, rating of family's standard of living within home: high = "many" or "some comforts," and low = "poor" or "just getting by".

[b]Academic performance: "During past 3 months, how well have you been doing with school work?": "better than average (grades of 8-10)"; "average (mostly grades of 7)"; "below average (mostly 5s and below)".

[c]High parental monitoring ("always" or "most of the time"); composite variable based on frequency of weekday & weekend monitoring students reported a lower prevalence of high parental monitoring ($p < 0.05$) (see Table 1). The risk behavior variables shared low to moderate levels of association with one another, with phi coefficients ranging from –0.02 to 0.36. Regression analyses showed that students reporting low parental monitoring were between 2 and 3.5 times more likely to report the six risk behaviors examined (see Table 2).

### DISCUSSION

This study found that Salvadoran youth with parents who know their whereabouts during the weekday and weekend were less likely to report substance use, aggression, and sexual risk behavior. These findings contribute to a growing body of evidence in support of parental monitoring[3,4,5,8] and suggest that parental monitoring is also an important factor for understanding risk behavior engagement among youth in El Salvador.

**Table 2.** Odds ratios for selected health risk behaviors by perceived parental monitoring in public secondary school students from central El Salvador, adjusted for socio-demographic variables, 1999. (n = 935)

| | Binge drinking[d,e] | Lifetime cig. use[d,f] | Illicit drug use[d,g] | Physical fight[d,h] | Sex. intercourse[d,i] | No condom use[d,j] |
|---|---|---|---|---|---|---|
| | Exp(B) 95% CI | Exp(B) 95% CI | Exp(B) 95% CI | Exp(B) 95% CI | Exp(B) 95% CI | Exp(B) 95% CI |
| Parental monitoring[a] | 3.22 (1.70, 6.11) | 2.03 (1.20, 3.43) | 3.51 (1.78, 6.96) | 2.81 (1.67, 4.73) | 3.25 (1.80, 5.88) | 3.32 (1.20, 9.17) |
| Gender (ref. = female) | 1.06 (0.62, 1.79) | 3.06 (2.16, 4.33) | 1.96 (1.08, 3.57) | 2.62 (1.86, 3.70) | 10.19 (6.41, 16.20) | 1.28 (0.49, 3.37) |
| Age (12-20 years) | 1.15 (0.95, 1.38) | 1.10 (0.97, 1.24) | 0.92 (0.75, 1.13) | 0.96 (0.85, 1.09) | 1.44 (1.25, 1.65) | 1.03 (0.81, 1.31) |
| Subj. econ. status[b] (ref. = high) | 1.32 (0.77, 2.25) | 0.91 (0.63, 1.29) | 1.44 (0.80, 2.58) | 1.22 (0.86, 1.73) | 0.81 (0.55, 1.21) | 1.02 (0.49, 2.11) |
| Academic performance[c] | 2.00 (1.27, 3.16) | 1.45 (1.07, 1.97) | 1.52 (0.94, 2.48) | 1.96 (1.45, 2.66) | 1.18 (0.86, 1.63) | 0.77 (0.43, 1.35) |
| Geographic region (ref. = rural) | 1.90 (1.08, 3.36) | 2.08 (1.18, 3.69) | 1.23 (0.58, 2.62) | 2.54 (1.64, 3.94) | 1.24 (0.83, 1.86) | 1.02 (0.38, 2.71) |

Odds ratios calculated using gllamm function of Stata to adjust for nesting of students within schools. Results based on simultaneous regression analysis.

Abbreviations: ref, referent group; Subj. econ. status, subjective economic status; CI, Confidence Interval; Cig., Cigarette.

[a]Dichotomous composite variable (coded 0,1) of the frequency of weekday & weekend monitoring. Referent group = high parental monitoring ("always" or "most of the time").

[b]Subjective economic status, rating of family's standard of living within home: high (referent group) = "many" or "some comforts," and low = "poor" or "just getting by".

[c]Academic performance: "During past 3 months, how well have you been doing with school work?": "better than average (grades of 8-10)"; "average (mostly grades of 7)"; "below average (mostly 5s and below)".

[d]All risk behavior variables were dichotomous (0,1), with 1 = having engaged in risk behavior.

[e]Having drunk 5 or more alcoholic beverages in a row on 1 or more of the 30 days preceding the survey.

[f]Having ever tried cigarette smoking.

[g]Having ever used marijuana, inhalants, or cocaine.

[h]Having participated in a physical fight ≥1 time in the past 12 months.

[i]Having ever had sexual intercourse.

[j]Did not use a condom (partner or self) during last sexual intercourse.

Consistent with findings from the U.S.[3,4], we found girls to report higher prevalence of parental monitoring. Although the overall evidence in the published literature for the parental monitoring-risk behavior association appears strong, some studies have found the association to differ by gender[8] or have found inconsistent associations for Hispanics living in the U.S.[9]. Our findings suggest a strong inverse association between selected youth risk behaviors and perceived parental monitoring in Salvadoran youth that persists after adjusting for gender, geographic status, and other sociodemographic factors.

Some limitations should be considered when interpreting the results of this study. First, as conditions in El Salvador prevent 50% of adolescents from attending secondary school[10], these results may not be generalizable to those adolescents who may be most at risk for engagement in health and social risk behaviors. Second, the study was based on two items that assessed parental monitoring. As some parental monitoring measures have included a dimension of child-parent communication[4,8], findings of the present study may have underestimated the association between parental monitoring and engagement in youth risk behaviors. Lastly, this study is based on a cross-sectional design, which precludes inferences on causality.

Recognizing these limitations, our findings suggest that developing and evaluating intervention strategies to promote parental monitoring may hold promise for reducing risk behavior of Salvadoran youth.

### ACKNOWLEDGMENTS

The authors thank the National Cancer Institute pre- and postdoctoral fellows at the University of Texas School of Public Health who helped review drafts of this manuscript, as well as the staff at the Centro de Capacitación of the Ministry of Education of El Salvador for their logistical support of the original study. Preparation of this manuscript was made possible in part by National Cancer Institute/NIH Grant #2R25CA57712 supporting Dr. Springer's Postdoctoral Fellowship, Cancer Prevention and Control Training Program in the Center for Health Promotion and Prevention Research, University of Texas-Houston School of Public Health.
